# A diagnostic dilemma between psychosis and post-traumatic stress disorder: a case report and review of the literature

**DOI:** 10.1186/1752-1947-5-97

**Published:** 2011-03-10

**Authors:** Ricardo Coentre, Paddy Power

**Affiliations:** 1 Lambeth Early Onset (LEO) Service, Lambeth Hospital, South London and Maudsley NHS Trust, 108 Landor Road, London SW9 9NT, UK; 2 Institute of Psychiatry, King's College, 16 De Crespigny Park, London SE5 8AF, UK; 3 Department of Psychiatry, Faculty of Medicine, Hospital Santa Maria, University of Lisbon, Av. Prof. Egas Moniz, 1649-035 Lisboa, Portugal

## Abstract

**Introduction:**

Post-traumatic stress disorder is defined as a mental disorder that arises from the experience of traumatic life events. Research has shown a high incidence of co-morbidity between post-traumatic stress disorder and psychosis.

**Case presentation:**

We report the case of a 32-year-old black African woman with a history of both post-traumatic stress disorder and psychosis. Two years ago she presented to mental health services with auditory and visual hallucinations, persecutory delusions, suicidal ideation, recurring nightmares, hyper-arousal, and initial and middle insomnia. She was prescribed trifluoperazine (5 mg/day) and began cognitive-behavioral therapy for psychosis. Her psychotic symptoms gradually resolved over a period of three weeks; however, she continues to experience ongoing symptoms of post-traumatic stress disorder. In our case report, we review both the diagnostic and treatment issues regarding post-traumatic stress disorder with psychotic symptoms.

**Conclusions:**

There are many factors responsible for the symptoms that occur in response to a traumatic event, including cognitive, affective and environmental factors. These factors may predispose both to the development of post-traumatic stress disorder and/or psychotic disorders. The independent diagnosis of post-traumatic stress disorder with psychotic features remains an open issue. A psychological formulation is essential regarding the appropriate treatment in a clinical setting.

## Introduction

Post-traumatic stress disorder (PTSD) is defined as a mental disorder that arises from the experience of traumatic life events. Documented symptoms include re-experiencing the traumatic event, hyper-arousal and avoidance of stimuli associated with the trauma [1]. None of the Diagnostic and Statistical Manual of Mental Disorders Text Revision (DSM-IV-TR) diagnostic criteria refers to psychotic phenomena such as delusions or hallucinations. Research has shown a high incidence of co-morbidity between PTSD and psychosis; for example, psychosis with PTSD and *vice versa *[2]. The emergence of psychosis in PTSD raises important nosological questions about the disorder. In our case report, we describe the case of a patient with PTSD who later developed psychotic features. We will also discuss and review the nosological and treatment implications of this co-morbidity. To the best of our knowledge, we report the first case of PTSD with psychotic symptoms in a pregnant woman treated with trifluoperazine.

## Case presentation

We present the case of a 32-year-old black African, muslim woman with a history of both PTSD and psychosis. She presented to mental health services for the first time two years ago with a history of auditory and visual hallucinations, persecutory delusions, suicidal ideation, recurring nightmares, hyper-arousal, and initial and middle insomnia. She reported seeing blood on the walls, men in white following her and hearing voices saying that some men were coming to get her. These symptoms were worse at night. She became very distressed and troubled to the point of wanting to end her life.

Her background history suggested co-morbid PTSD. Twelve years ago, she saw her family (parents, sisters and brother) being killed during the civil war in her birth country in Africa. Her clinical PTSD symptoms, such as the recurring nightmares, hyper-arousal, and initial and middle insomnia, began shortly afterwards. Eight years later, she came to the UK as an asylum seeker. During her first few years in the UK, she had no social support, was unable to speak English, experienced homelessness and was unsuccessful in gaining asylum. Her auditory and visual hallucinations and persecutory delusions started at this time. A few months before her first contact with mental health services, her psychotic symptoms and PTSD features became more frequent and intense. With no stable relationship she became pregnant and visited her general practitioner who referred her to our first-episode psychosis unit.

Upon admission, she presented as well kempt yet she appeared distressed. She was withdrawn and quiet and there was some delay in her responses to questions. She was tearful and her mood was low but reactive. She described vivid and clear auditory and visual hallucinations and persecutory delusions. Her medical psychiatric, personal, and family histories were unremarkable. A physical examination, neurological examination and brain magnetic resonance imaging (MRI) scan were normal. The results of our routine blood investigations were in the normal range, and a pregnancy test was positive. At our clinical interview, she clearly fulfilled the DSM-IV-TR criteria for PTSD and psychotic disorder not otherwise specified (NOS).

Because of the intensity of her symptoms, her distress and suicidal ideation, our mental health team recommended ongoing hospitalization. She was started on trifluoperazine (5 mg/day) and cognitive-behavioral therapy for psychosis. She also started a prenatal follow-up. She self-reported a partial improvement in her clinical picture and her psychotic symptoms gradually resolved over a three-week period, although they occasionally resurfaced when she was under stress or whenever her medication compliance lapsed. She was discharged from hospital and is now living in temporary accommodation funded by local services and waiting for her asylum re-application to be processed. She continues to have ongoing PTSD symptoms associated with the initial tragic event as persistent remembering of the stressor event with recurring and vivid memories, nightmares, hyper-arousal and initial insomnia. She also avoids circumstances resembling the initial stressor event, such as wars and violence.

## Discussion

In our case report, we describe the case of a patient with PTSD with psychotic symptoms. Her PTSD developed soon after a severe traumatic experience associated with a civil war twelve years ago: she witnessed the murder of her nuclear family. Eight years later she developed psychotic symptoms, which included auditory and visual hallucinations and persecutory delusions. She finally presented two years ago to mental health services in the context of major social stresses, an unwanted pregnancy, potential homelessness, and a rejected asylum claim. Symptomatically, her psychosis responded well to treatment but the PTSD features and stresses remain. Her follow-up is now directed towards dealing with these issues, as well as preventing a relapse.

We reviewed the scientific literature regarding the diagnosis and treatment of PTSD with psychotic symptoms. There are few case reports about the presence of PTSD with psychotic features, mainly involving war veterans, but none using trifluoperazine as a psychopharmacological treatment. In 2008, Floros *et al. *reported the case of a man with psychotic symptomatology after a traumatic event involving the accidental mutilation of his fingers. His treatment plan included pharmacotherapy and supportive psychotherapy with the establishment of a good doctor-patient relationship. This biopsychosocial approach was made to integrate all aspects relating to his history in a meaningful way [3].

In our case report, our patient had PTSD symptoms including experiencing recurrent distressing images of the traumatic event, with a markedly diminished interest and participation in significant activities and the avoidance of thoughts and conversations associated with the trauma. She also had persistent symptoms of increased arousal, with difficulty falling and staying asleep. PTSD with psychotic symptoms is associated with a clinically significant impairment in social and occupational functioning, including difficulties in getting a stable job and holding down relationships. According to the DSM-IV-TR, PTSD is classified as an anxiety disorder but expressions of the disorder may include obsessions, phobias, dissociations or depression [4]. Less characteristic and poorly studied, are the psychotic symptoms associated with PTSD. Our patient presented with visual and auditory hallucinations and persecutory delusions with content that mirrored her PTSD. In patients who do not have another established severe mental illness, the presence of psychotic symptoms in PTSD might be better captured as a dimension or sub-group of PTSD rather than psychosis NOS.

Mueser *et al. *have suggested that PTSD influences psychosis both directly, through the effects of specific PTSD symptoms including avoidance, over-arousal and re-experiencing the trauma, and indirectly, through the effects of common consequences of PTSD such as re-traumatization, substance abuse and difficulties with interpersonal relationships [5]. Our patient had both; she frequently "relived" the traumatic event through intrusive flashbacks and recurring dreams. Co-morbid psychosis has been described in approximately 20 to 40 percent of veterans with combat-related PTSD [6,7]. The prevalence of PTSD in patients with a severe mental illness is at least three times higher (29 percent) than the general population [5]. In PTSD, the psychotic symptoms may be more pervasive or frequent than psychotic-like symptoms that occur during dissociative episodes or flashbacks [8]. PTSD with psychotic symptoms has also been reported in non-combat related cases of patients with PTSD but not schizophrenia-spectrum or bipolar disorders.

From a psychological point of view, there is a relationship between the individual's pre-existing cognitive schemas and thought patterns emerging after the traumatic event. A maladaptative cognitive processing style culminates in feelings of shame, guilt and worthlessness, which emerge during trauma acting as positive feedback to enhance symptom severity and keep the individual in a constant state of psychotic turmoil. It is possible that under certain individual-specific conditions, the defence and coping mechanisms break down at a level of psychotic manifestations in the form of delusions and hallucinations. It has been hypothesized that trauma may produce a psychological vulnerability leading to the development of psychotic experiences. In our patient, factors such as an unwanted pregnancy, potential homelessness and a rejected asylum claim may have contributed to and triggered the emergence of psychotic features in a preceding PTSD. Some authors underline the importance of both disorders being characterized by intrusions. In PTSD, the interpretation of intrusive symptoms such as flashbacks is seen as central to the maintenance of the disorder. In psychosis, hallucinations and delusional beliefs are interpretations of intrusions [9].

Unlike our case report, where there was clear evidence of a life-threatening trauma before psychotic symptoms, some authors identify psychosis itself as the source of trauma for patients with both conditions. There is some evidence suggesting that psychosis, hospitalization, or both may be sufficiently severe to precipitate PTSD and that psychological distress related to a psychotic episode may predict an evolution to PTSD [10].

Our patient was an immigrant from a black ethnic minority group. First- and second-generation black ethnic minority migrants are at a particularly high risk of psychosis in London. The explanation for these findings is uncertain, but social adversity, racial discrimination, family dysfunction, unemployment, poor housing conditions and urbanicity have been proposed as contributing factors [11-13]. It is possible that similar stresses contributed to the heightened risk of psychosis in our patient.

Some authors argue for a new condition called PTSD with psychotic symptoms, claiming that it should be included in the psychiatric classification systems to account for the high percentage of psychotic symptoms in patients with PTSD [14]. Our patient could fit into this category.

Establishing a correct diagnosis is imperative in developing an appropriate treatment strategy, particularly when the presence of psychotic symptoms necessitates the use of anti-psychotic medication. In addition to the demonstrated efficacy of selective serotonin re-uptake inhibitors (SSRIs), a range of other drugs, including second-generation anti-psychotics, have recently been investigated for the treatment of PTSD. The currently available evidence suggests that first-line pharmacotherapy is SSRIs and possibly the serotonin norepinephrine re-uptake inhibitor venlafaxine extended release [15]. Response rates are limited: approximately 60 percent of patients treated with SSRIs are reached [16]. Psychotic symptoms are associated with more severe symptomatology and their presence is also known to decrease the efficacy of conventional treatment [17], further indicating a possible role for an anti-psychotic treatment. We found a paucity of randomized, double-blind, placebo-controlled clinical trials (RCT) of anti-psychotics for the treatment of PTSD. However case reports, small RCTs and open-label studies have demonstrated the beneficial effect of this pharmacotherapy (add-on and monotherapy) for the treatment of PTSD patients with and without psychotic symptoms. Published case reports demonstrate the efficacy of clozapine [8] or amisulpride [3] in the treatment of both PTSD and psychotic symptoms. Fluphenazine, olanzapine, risperidone and quetiapine are anti-psychotics with demonstrated efficacy in open clinical trials as a monotherapy in PTSD with psychotic features [18-20].

Hamner described the case of a Vietnam veteran with a history of PTSD symptoms and psychotic symptoms including auditory hallucinations, visual hallucinations, thought disorder and paranoid ideation. He had a history of substance abuse (alcohol and cocaine) but had been in remission for one year prior to his evaluation. He was treated unsuccessfully with typical neuroleptics, electroconvulsive therapy, benzodiazepines and lithium. Clozapine was initiated and titrated to 600 mg/day leading to an improvement of his PTSD and psychotic symptoms [8].

However, to date, none of these agents has received registration status for use in PTSD in the USA or in Europe [21]. In the absence of guidelines relating to the condition of PTSD with psychosis, our patient's psychosis responded well to the standard anti-psychotic treatment but her co-morbid PTSD features remain. Given her complicated presentation, her recovery will require a multi-faceted approach with an emphasis on addressing her pre-existing PTSD. She did not develop any extra-pyramidal symptoms associated with the use of a typical anti-psychotic, however, Chan *et al. *report the cases of three patients with PTSD with psychotic features who developed severe extra-pyramidal side effects, namely akathisia, leading to the withdrawal of the anti-psychotic medication [22].

Several psychotherapeutic interventions have been studied in PTSD and psychotic illnesses, with a growing literature suggesting that they are both feasible and effective. Waldfogel *et al. *report the case of a non-combat veteran with PTSD with psychotic symptoms who was not successfully treated with anti-psychotics and for whom exposure therapy was successful in treating PTSD and psychosis [23]. Mueser *et al. *published a randomized controlled trial of the cognitive-behavioral treatment (CBT) of PTSD in severe mental illness, which includes breathing retraining, education about PTSD and cognitive restructuring. Results indicated that patients included in a 12- to 16-session CBT program showed a greater improvement of their PTSD symptoms, other symptoms, perceived health, negative trauma-related beliefs, knowledge about PTSD, and case manager working alliance compared with treatment as usual, where patients continued to receive the usual treatments they had been undertaking in local mental health centers [24]. Frueh *et al. *report an open trial in adults with PTSD and either schizophrenia or schizoaffective disorder who were treated via an 11-week cognitive-behavioral intervention. The trial involved 22 group and individual sessions for PTSD consisting of anxiety management therapy, psycho-education, social skills training and exposure therapy. Participants showed a significant improvement of their PTSD symptoms and high treatment satisfaction [25]. Besides the psychopharmacological therapy, our patient could benefit from one of these psychotherapeutic programs targeting PTSD symptoms.

As in the case report published by Waldfogel *et al.*, patients presenting with PTSD with psychotic features who do not have a well established severe mental illness might also respond to conventional psychotherapeutic treatments with a demonstrated efficacy for the treatment of PTSD in the general population [23]. Due to the paucity of published systematic studies, this is a field for future research.

Because our patient has no friends or family in the UK, our diagnosis was based only on self-reported information; a less rigorous approach than those using other sources of information to corroborate a patient's account. A structured clinical interview and the use of specific measure instruments could also help in rating symptoms and promoting an improvement in clinical daily routine.

## Conclusions

There are many factors responsible for the symptoms that occur in response to a traumatic event, including cognitive, behavioral, physiological, affective and environmental factors. These factors may predispose to the development of PTSD and/or psychotic disorders. The independent diagnosis of PTSD with psychotic features remains an open issue. Evidence seems to demonstrate that the two disorders - PTSD and psychosis - may both emerge from a traumatic experience, or that PTSD itself may increase the risk of subsequent psychotic illness. A psychological formulation addressing the potential causes of PTSD and psychosis that could be treated with specific interventions (such as CBT) is essential.

## Abbreviations

CBT: cognitive-behavioral therapy; DSM-IV-TR: diagnostic and statistical manual of mental disorders, fourth edition, text revision; MRI: magnetic resonance imaging; NOS: not otherwise specified; PTSD: post-traumatic stress disorder; RCT: randomized, double-blind, placebo-controlled clinical trials; SSRIs: selective serotonin re-uptake inhibitors.

## Competing interests

The authors declare that they have no competing interests.

## Authors' contributions

RC designed the study, reviewed the existing literature and drafted the manuscript. PP carried out the follow up on the patient, took part in the scientific discussion and helped to draft the manuscript. All authors read and approved the final manuscript.

## Consent

Written informed consent was obtained from the patient for publication of this case report and any accompanying images. A copy of the written consent is available for review by the Editor-in-Chief of this journal.
